# Auto encoder-based defense mechanism against popular adversarial attacks in deep learning

**DOI:** 10.1371/journal.pone.0307363

**Published:** 2024-10-21

**Authors:** Syeda Nazia Ashraf, Raheel Siddiqi, Humera Farooq

**Affiliations:** Department of Computer Science, Bahria University, Karachi, Pakistan; University of Manitoba, CANADA

## Abstract

Convolutional Neural Network (CNN)-based models are prone to adversarial attacks, which present a significant hurdle to their reliability and robustness. The vulnerability of CNN-based models may be exploited by attackers to launch cyber-attacks. An attacker typically adds small, carefully crafted perturbations to original medical images. When a CNN-based model receives the perturbed medical image as input, it misclassifies the image, even though the added perturbation is often imperceptible to the human eye. The emergence of such attacks has raised security concerns regarding the implementation of deep learning-based medical image classification systems within clinical environments. To address this issue, a reliable defense mechanism is required to detect adversarial attacks on medical images. This study will focus on the robust detection of pneumonia in chest X-ray images through CNN-based models. Various adversarial attacks and defense strategies will be evaluated and analyzed in the context of CNN-based pneumonia detection. From earlier studies, it has been observed that a single defense mechanism is usually not effective against more than one type of adversarial attack. Therefore, this study will propose a defense mechanism that is effective against multiple attack types. A reliable defense framework for pneumonia detection models will ensure secure clinical deployment, facilitating radiologists and doctors in their diagnosis and treatment planning. It can also save time and money by automating routine tasks. The proposed defense mechanism includes a convolutional autoencoder to denoise perturbed Fast Gradient Sign Method (FGSM) and Projected Gradient Descent (PGD) adversarial images, two state- of-the-art attacks carried out at five magnitudes, i.e., ε (epsilon) values. Two pre-trained models, VGG19 and VGG16, and our hybrid model of MobileNetV2 and DenseNet169, called Stack Model, have been used to compare their results. This study shows that the proposed defense mechanism outperforms state-of-the-art studies. The PGD attack using the VGG16 model shows a better attack success rate by reducing overall accuracy by up to 67%. The autoencoder improves accuracy by up to 16% against PGD attacks in both the VGG16 and VGG19 models.

## Introduction

Pneumonia, an infection affecting the lungs, can impact individuals across all age groups, particularly those with weakened immune systems. Its prevalence is notably higher among children under the age of 5. It is also common in old age (above 60 years). According to the United Nations Children’s Fund (UNICEF), pediatric pneumonia claims the lives of over 800,000 young children worldwide each year, illustrating the mortality rate associated with pneumonia in children [1–3]. Because of the higher death rate in children, this research will focus on the diagnosis of pediatric pneumonia detected in Chest X-ray images. If pneumonia is suspected, the doctor may recommend different tests. Typically, a chest X-ray examination is a simple, rapid, cost-effective, and relatively safe procedure with minimal radiation exposure risk [4]. Many clinical illnesses can be evaluated by X-rays in which pneumonia is one of them. In third-world countries, an X-ray is the most economical test, and it is readily available for diagnosis. For this reason, the chest X-ray modality is selected for the diagnosis of pneumonia.

Medical image analysis plays a crucial role in reducing medical errors, which is vital given that a study [5] has identified medical errors as the third leading cause of death in the USA. Additionally, research [6] indicates that the advancement of medical imaging contributes to the increase in human life expectancy. An additional perspective is presented by Beinfeld et al. [7] who declared that approximately $3000 can be saved by spending $385 on medical imaging. Frequently utilized imaging techniques encompass X-ray, Magnetic Resonance Imaging (MRI), Computed Tomography (CT) scans and Ultrasound (US). Yet, due to the difficulty in obtaining medical images, which often result in smaller datasets compared to other computer vision tasks, transfer learning is frequently employed [8]. Medical image analysis encompasses various tasks, with classification or diagnosis, detection, and segmentation being among the most significant deep learning tasks in this field.

In this research work, pediatric pneumonia will be detected through medical image classification using pediatric Chest X-ray images that is publicly available at [9]. The primary goal of medical image classification has two aspects: achieving high accuracy and pinpointing the affected regions of the human body by the disease [10]. Deep learning algorithms, notably Convolutional Neural Networks (CNNs), have emerged as a preferred method for analyzing medical images due to their ability to effectively learn significant features. They progressively extract higher- and higher-level representations of the image content [11]. While deep learning algorithms have demonstrated remarkable performance in medical image classification, security emerges as a primary concern due to their susceptibility to manipulation by adversarial examples. By introducing subtle alterations to pixel values in an image, known as perturbations, attackers can prompt a convolutional neural network (CNN) to change its prediction, potentially leading from a classification of "Normal" to "Bacterial Pneumonia". These adversarial perturbations, essentially layers of imperceptible noise, can deceive the model’s predictions. Despite the seeming impracticality of adversarial examples in medical image analysis, it’s crucial to consider potential motivations behind such attacks. Given the substantial financial support within the healthcare economy, there exists a risk that malicious actors may exploit vulnerabilities to profit through manipulation of the healthcare system. For instance, attackers could manipulate test reports to commit insurance fraud or submit false claims for medical reimbursement [12]. Moreover, an inaccurate diagnosis can result in harmful effects on the patient’s life, potentially leading to unnecessary costs and utilization of medical resources [13]. Furthermore, some malicious consultants may exploit these attacks to increase their profits by compromising the integrity of confidential information through the alteration of test reports, resulting in unnecessary surgical procedures. Alternatively, an attacker might aim to disrupt operations by covertly manipulating an image to induce a misdiagnosis of a disease, potentially leading to significant repercussions in patient care [14]. This illustrates hypothetical scenarios wherein attackers could create adversarial examples. For instance, consider a clinic that could manipulate medical images to prompt unnecessary surgical interventions. Consequently, the safety implications of deep neural networks have become a significant area of concern.

Deep neural networks are susceptible to adversarial attacks, wherein subtle perturbations are introduced to inputs, resulting in erroneous predictions with high confidence. These perturbations, although often imperceptible to the human eye in the case of images, can profoundly deceive deep learning models, posing a significant threat to their effectiveness. Consequently, adversarial attacks present a serious challenge to the practical application of deep learning. This realization has sparked a considerable influx of research contributions in this area [15].

Szegedy C et al. [16] pioneered the investigation of adversarial attacks and it claimed that the efficacy of this attack is because of the absence of generalization in the low likelihood space of data. However, subsequent research [17,18] has shown that linear models are also susceptible as well and a raise in the model’s capacity improves resilience against these attacks. The research [19] suggests that it is significant to study the existence of adversarial examples and understand deep learning algorithms to create more robust models.

Adversarial training stands out as one of the most widely adopted defense strategies, enhancing model robustness by incorporating adversarial samples during training. While effective, adversarial training still faces susceptibility to black-box attacks, limiting its efficacy to the types of attacks in which they are trained. In a study [20], ensemble adversarial training has been implemented as another potent technique, yielding models that exhibit strong resilience against black-box attacks. Neural networks are trained using adversarial samples generated from techniques like FGSM and PGD, thereby diversifying the training dataset. Another defense mechanism, randomization [21], aims to introduce randomness into adversarial samples. Denoising on the other hand attempts to remove perturbations from inputs [22]. Additionally, defenses such as weight-sparse Deep Neural Networks (DNNs) [23], k-nearest neighbor algorithm (KNN)-based defenses [24], Bayesian model- based defense [25], and consistency-based defenses [26] have been proposed. Conversely, detection methods also play a vital role, identifying adversarial samples and discarding them before inputting them into the classifier [26,27].

An engineered adversarial attack can compromise a pneumonia detection model based on chest X- rays (CXR) with subtle perturbations that are barely noticeable.

An effective and generic defense mechanism is necessary to safeguard CXR-based pneumonia detection systems against such adversarial attacks. The contributions of this paper are as follows:

1. Develop a robust combined model for detecting bacterial and viral pneumonia in chest x-ray images through deep learning that is resilient against state-of-the-art FGSM and PGD adversarial attacks [28,29] and predicts the correct output.
2. Design and evaluate an auto-encoder-based defense technique that can detect and defuse state-of- the-art FGSM and PGD adversarial attacks.
3. To improve classification accuracy of reconstructed images generated by auto-encoder up to the acceptable level.

The structure of the remaining paper is as follows. Literature review presents existing adversarial attacks and defenses and detection methods on medical images. The methodology section presents the techniques and the chest X-ray dataset exploited in the research work undertaken. The results section presents the results obtained. The section also analyzes the results. Finally, the last section briefly discusses the key findings and limitations of the research study along with some associated directions for future work.

## Literature review

The domain of adversarial attacks is relatively new within the domain of medical image analysis. A recent study explored the applications, techniques, and limitations in this area [30]. Previous research has observed that many existing studies aim to demonstrate the impact of adversarial attacks on medical images through the utilization of established attack methods. However, some researchers propose new attacks or defensive measures to counter these attacks in medical images. A survey conducted to investigate the datasets used for adversarial attacks [31]. This survey shows that most of the existing work is tested with MRI, X-rays, and dermoscopy images. This survey also concluded that the gradient-based attacks i.e. FGSM, PGD, I-FGSM, etc. are the most effective, and for that reason, they are frequently used. Moreover, most new attacks are compared with these. Existing studies predominantly focus on two tasks: classification and segmentation. Moreover, most of the literature utilizes pre-trained models for evaluations. These models were originally designed for natural images, which are inherently more complex and thus require a larger number of parameters. However, models tailored for medical images typically necessitate fewer parameters. As noted by [32], over-parameterization of these models could significantly contribute to a substantial decrease in accuracy. U-Net emerges as the most utilized model for segmentation tasks, given its state-of-the-art performance in this domain, whereas ResNets are frequently employed for classification tasks. DenseNets are perceived as the most robust pre- trained models, with dense blocks enhancing the model’s security [31].

Ma X et al. [32] evaluate ResNet50’s resilience across three datasets (chest X-ray, ISIC, and fundoscopy) against four well-known untargeted attacks: FGSM, PGD, Carlini and Wagner (C&W), and Basic Iterative Method (BIM). They apply four detectors ((Kernel Density Estimation (KD), Local Intrinsic Dimensionality (LID), deep features, and quantized deep features-based detectors), achieving very high detection accuracy for identifying adversarial samples.

Researchers in [14] employed PGD white and black-box attacks using a pre-trained ResNet50 model on fundoscopy, dermoscopy, and chest X-ray images, resulting in a significant reduction in model accuracy in both scenarios. [33] investigated the classification accuracy of COVID-19 using X-rays and CT scans, applying the FGSM attack to generate adversarial samples. These samples were then tested on VGG-16 and InceptionV3 models, revealing a reduction in accuracy of up to 90% in VGG-16 and up to 63% in InceptionV3, highlighting the vulnerability of these models. A noteworthy study [34] showcased that self-supervised learning (SSL) models outperform ImageNet-based transfer learning (TL) models by learning more robust features. Additionally, [35] examined the susceptibility of a pediatric pneumonia detection model to PGD attacks. They found that increasing ε from 0.0001 to 0.009 led to a sharp degradation in diagnostic performance, although the performance remained relatively stable thereafter. Notably, the PGD attack was observed to have a more detrimental effect on the specificity of the model than its sensitivity.

Researchers in [36] examines the impact of biomedical image types, control parameters, and dataset size on the success of the PGD adversarial attack. Using chest X-ray and histology images with the Inception V3 model across eight classification tasks, they find that histology images are less vulnerable. Additionally, high-confidence original classifications reduce attack accuracy. Increasing perturbation generally increases attack success, except for geometrically distinct pathological changes like interior rotation in aorta X-ray images. Surprisingly, the study shows that training set size does not affect attack success.

Meanwhile, Asgari Taghanaki S et al. in [37] attempted to replace max pooling with average pooling. They crafted adversarial examples using the InceptionResNetV2 and Nasnet Large models on a chest X-ray dataset, employing ten different attacks categorized into gradient-based, score- based, and decision-based attacks. Results demonstrated that gradient-based attacks effectively deceived the models even with average pooling, while providing improvements in score-based and decision-based attacks.

A study examined several COVID-19 diagnostic methods employing deep learning (DL) algorithms alongside relevant adversarial examples (AEs) such as FGSM, MI-FGSM, Deepfool, L-BFGS, C&W, BIM, Foolbox, PGD, and JSMA [38]. Test outcomes highlighted that DL models lacking defensive mechanisms against adversarial perturbations remained susceptible to such attacks. The author introduced numerous novel passive and active attacks on Deep Neural Networks (DNNs), developed and evaluated across medical datasets [39]. Their new attacks reveal a largely under-explored attack surface of DNN inference engines. Two passive attacks can steal the valuable IP of the DNN models. The threat of active attacks in medical applications is demonstrated by an adversarial attack and a fault injection attack. In another investigation, the susceptibility of five commonly employed neural networks—specifically ResNet-18, ResNet-50, Wide ResNet-16-8 (WRN-16-8), VGG-19, and Inception v3—to adversarial attacks was examined [40]. Four distinct adversarial attack techniques, including the Fast Gradient Sign Method (FGSM), Projected Gradient Descent (PGD), Carlini and Wagner (C&W), and Spatial Transformations Attack (ST), were utilized for this assessment. Results indicated that ResNet-50 and WRN-16-8 exhibited comparatively lower vulnerability to these adversarial attacks.

Table 1 provides an overview of adversarial attacks applied to medical images. The column on performance decline illustrates that certain attacks can significantly reduce model accuracy, particularly in classification tasks. Among these attacks, FGSM and PGD were predominantly utilized, with PGD demonstrating superior effectiveness. Furthermore, the majority of experiments were performed using X-ray images.

**Table 1:** Summary of adversarial attacks on medical images.

Ref	Attack Method	Models	Data Modality	Performance Decline (%)
[35], 2023		VGG16	X-ray	The lowest accuracy attained is 33.33% (Acc drop 62.82). Attack success Rate is 66.66%
[36],	PGD	Inception V3	X-ray,	Upto 100% acc drop
2019			Histology	
[14], 2018		ResNet 50	Fundoscopy, Dermoscopy, X-ray	From 50–100% acc drop
[34],		VGG11, UNet	X-ray, MRI	Upto 100% acc drop
2020	FGSM, PGD			
[39],		ResNet-18	X-ray	Best Attack performance is
2020				94.7%
[32], 2021	FGSM, C&W, PGD, BIM	ResNet 50	X-ray, Dermoscopy Fundoscopy	Upto 100% acc drop
[33], 2021	FGSM	VGG16, Inceptionv3	CT-scan, X-ray	X-ray: accuracy decreased to 7.41%; CT-scan: accuracy decreased to 1.33%. Best Attack performance is 83.3%
[38], 2020	FGSM,MIFGS M, DF, LBFGS, C&W,BIMFB, PGD, JSMA BD, S, poisoning	ResNet, YOLO, DarkNet, GRAD-CAM	CT-scan X-ray	Best Attack performance is 91%
[40], 2021	FGSM,PGD, C&W, ST	ResNet50, ResNet18 WRN-16-8 VGG- 19,InceptionV3	X-ray	Avg Acc drop 25.5% to 56.3%
[37], 2018	FGSM, PGD, BIM, L-BFGS, DeepFool	NesnetLarge, InceptionResNet V2		White box: accuracy 0%, AUC 0; Black box: accuracy 51%, AUC 0.49

A study [41] explores the adversarial bias field attack, which employs bias fields instead of additive noise to deceive DNNs. To address this challenge, they propose the adversarial-smooth bias field attack, which locally adjusts the bias field with joint smooth and adversarial constraints. Evaluation on chest X-ray datasets with ResNet50, MobileNet, and DenseNet121 models shows its superior attack accuracy in terms of transferability compared to other well-known attacks.

The study [42] presents a hierarchical feature constraint (HFC) approach to evade detection in adversarial attacks. This method aims to obscure adversarial features, making detection more challenging, particularly in medical images. Evaluation with X-ray and fundoscopy images using ResNet50 and VGG16 models demonstrates its effectiveness.

The studies [43,44] have identified adversarial samples across numerous images, which were then applied to medical datasets in [32]. In another study [45], a defense strategy was proposed for classification, segmentation, and object detection challenges using a non-linear radial basis convolutional feature mapping approach. By learning a Mahalanobis-like distance function, it addresses the linear and inflexible nature of deep learning models. Evaluation on NIH Chest X- ray 14 dataset and ISBI ISIC 2017 dataset shows improved accuracy for both clean and perturbed images in classification and segmentation tasks. [45] proposes a defense strategy for classification, segmentation, and object detection challenges using a non-linear radial basis convolutional feature mapping approach. By learning a Mahalanobis-like distance function, it addresses the linear and inflexible nature of deep learning models. Evaluation on NIH Chest X- ray 14 dataset and ISBI ISIC 2017 dataset shows improved accuracy for both clean and perturbed images in classification and segmentation tasks.

In another study [46], A novel Fuzzy Unique Image Transformation (FUIT) technique defends COVID-19 deep models against six adversarial attacks (FGSM, BIM, PGD without random start, PGD-r with random start, Deep Fool, C&W) by downsampling image pixels to an interval before training. It maintains high accuracy in distinguishing COVID-19 cases from chest X-ray and CT image datasets without altering model architecture. While it requires time-consuming image transformation during testing, it proves more effective than conventional discretization approaches in protecting models against attacks.

In [47], three pre-trained deep diagnostic models were evaluated for resilience against PGD and GAP attacks across various tasks: melanoma detection (IPMI2019-AttnMel), diabetic retinopathy detection (InceptionV3), and classifying 14 diseases on chest X-rays (CheXNet). Unfortunately, these models were unreliable against PGD and GAP attacks, with significant accuracy decreases even with 100% accuracy in PGD attacks. Two novel defense techniques were developed: multi- perturbations adversarial training (MPAdvT) involving training DNNs with varied perturbation levels and adversarial iteration steps, and a misclassification-aware regularization technique using Kullback-Leibler (KL) divergence. Experimental results showed superior performance compared to standard adversarial training methods.

The study [48], presents an attack detection method that identifies adversarial examples without prior knowledge of attackers, while maintaining classification performance. It seamlessly integrates into deep learning-based medical imaging systems, enhancing robustness. The method distinguishes clean and adversarial images based on high-level features, particularly during convolution-pooling operations in CNN models. Testing on the Chest X-ray 14 dataset with DenseNet121 confirms its efficacy, offering a significant advantage without requiring prior knowledge of attack methods or CNN architecture modification.

The authors propose MedRDF (Robust and Retrain-Less Diagnostic Framework for Medical pretrained models against adversarial attacks), which operates during the inference phase of pretrained medical models, in their study [49]. MedRDF generates noisy copies of test images and obtains their output labels from the pretrained model, then utilizes majority voting for final diagnosis. It also generates a Robust Metric (RM) for confidence assessment. MedRDF effectively enhances the robustness of clinical investigative models, as demonstrated on COVID-19 and DermaMNIST datasets, without requiring retraining.

Furthermore, a novel mechanism proposed by [50] that boosts the robustness of medical image classification systems by integrating denoising capabilities into CNN classifiers. Using a naturally embedded auto-encoder and high-level feature invariance, it effectively addresses general noises. This mechanism complements existing defense approaches and proves effective in comprehensive evaluations across two medical image classification tasks.

A model-agnostic explainability-based method for accurate detection of adversarial samples in Electronic Health Record (EHR) and chest X-ray (CXR) datasets has been proposed by [51]. The approach utilizes explainability techniques for anomaly detection, demonstrating generalization across different attack methods without needing retraining.

A novel model-based defense framework for medical images, enhancing Deep Neural Networks (DNNs) with pruning and attention mechanisms was proposed by [52]. Through ablation experiments, it shows that integrating these mechanisms effectively enhances model robustness. This approach is tailored to address the specific challenges posed by medical images, surpassing existing defense methods designed for natural images.

The resilience of DL models trained on diagnostic images against adversarial attacks investigated in [53]. They find that DL models trained without adversarial considerations exhibit instability to minor pixel-level changes, leading to accuracy declines. However, employing iterative adversarial training significantly improves model stability and resilience against such alterations.

In a study [54], researchers quantifies the impact of imperceptible adversarial perturbations on medical image diagnosis and identifies noise within images as a significant factor contributing to CNN susceptibility. They propose a defense approach by embedding sparsity denoising operators into CNNs, effectively preserving over 90% of original performance against various attacking methods across different medical image modalities.

Table 2 provides an overview of countermeasures and attack detection techniques concentrating on the classification task within the X-ray modality, specifically targeting FGSM and PGD attacks. Numerous investigations have been conducted using popular attacks such as FGSM, BIM, PGD, and Momentum Iterative Methos (MIM), yielding encouraging results. Some approaches exhibit significant defense capabilities against attacks, while others merely reduce attack effectiveness. Additionally, attackdetection methods demonstrate high accuracy in identifying adversarial samples.

**Table 2:** Summary of defense and attack detection methods.

Mod aliti es	Ref& Year	Attack Method	Models	Defense Method	Performance
X-Ray	[28], 2023	FGSM, GD	LeNet5, MobileNetv1, VGG16, InceptionV3, ResNet50	Adversarial Training	Accuracy reduced up to 12 to 92%. The best defense performance is 82% (PGD) to 84% (FGSM)
	[36], 2020	FGSM, PGD, DAA, MI-FGSM, DII-FGSM	InceptionV3	Adversarial Training, Pre- processing	The success of attacks drops when original confidence of predicting image class exceeds 0.95.
	[29], 2022	FGSM, GD	VGG19	HGD: Pre- processing	Accuracy improves 0 to 74% (white-box), -31% to 0% (black-box)
	[49], 2022	I-FGSM, PGD, C&W, SPSA, Rays	RsNet50, ResNet18, AG-Sononet-16	MedRDF: Pre- processing	Best defense performance is 91.4%
	[52], 2021	PGD, FGSM	ResNet34, Auto Encoder, kWTA,	Feature Enhancement	Achieve up to 77.18% defense rate for PGD
			InvGAN, Prune- ResNet34, BAM- ResNet34, SelfAttn- Resnet34, CBAM- ResNet34, Prune- CBAM-ResNet34		Attack and 69.49% for DeepFool attack.
	[37], 2018	FGSM, PGD, BIM, L-BFGS, DeepFool	NesnetLarge, InceptionResNetV2		Accuracy increased by up to 9%(white and black- box settings)
	[51], 2021	PGD, CW	MIMIC-III RETAIN, Henan-Renmin RETAIN, MIMIC- CXR Densenet121	Adversarial Detection	Improves adversarial detection by over 10% Best defense performance is 78 to 88%
	[48], 2020	FGSM, BIM, PGD, MI-FGSM	DeseNet121		Attack Detection up to 97.5% Accuracy
CT/ MRI/ X-ray	[53], 2022	FGSM, PGD, BIM	VGG16	Adversarial Training	Absolute accuracy improves from 3.7% to 5.9%
	[46], 2020	Deep Fool, FGSM, BIM C&W, PGD PGD-r	CNN	FUIT: Pre- processing	Best defense performance > 96% Accuracy reduced by only up to 2%
X-ray/Dermoscopy	[54], 2022	FGSM, PGD	ResNet50, ResNet50- D, ResNet50-A-D	Feature Enhancement	defend by retaining as much as over 90% of its original performance
	[50], 2019	FGSM, I- FGSM, CW	ResNet18, VGG-16		Best defense performance is 70.92%. Accuracy is reduced by up to 24%
	[45], 2019	FGSM, CW, PGD, BIM, GN, SPSA, MI-FGSM	Inception-ResNet-v2, U-Net and V-Net		Best defense performance is 65%. Accuracy is reduced by up to 29%
	[47], 2021	PGD, GAP	CheXNet, Inceptionv3, Custom CNN	Adversarial Training	Improvement in Standard Defense method (Adversarial Training) by up to 9%
	[32], 2021	FGSM, BIM, PGD, C&W [32, 43, 44]	ResNet 50	Adversarial Detection	Attack Detection up to 100% Accuracy (white- box settings)
	[43], 2017				
	[44], 2018				

Despite extensive research on adversarial examples for natural images, relatively fewer studies have been conducted on medical images. Many researchers perceive the generation of adversarial examples in medical images as particularly challenging. In medical image analysis, it should be assured about the accuracy of the algorithms, and it should tackle adversarial examples as they can cause catastrophic outcomes. There are some limitations and future directions in the discussed studies. For example, there is a need to investigate other attack types and attack-independent defense mechanism to secure pediatric pneumonia detection models from all types of adversarial attacks. Furthermore, given the multifaceted nature of adversarial examples, it’s possible to implement multiple defense strategies concurrently. Expanding the dataset used for training pretrained models, which enhances classification accuracy and mitigates vulnerability, can be explored across various settings.

Most of the surveys indicate many adversarial attacks and defense mechanisms, in which most of the researchers find vulnerability [33,38,55] and robustness [24,31,35,47,53,56] of the model in the effect of adversarial attacks and none of the countermeasure is a remedy for maximum categories of attacks i.e. all defense techniques reviewed are effective only for a particular category of adversarial attacks and are not capable to tackle all categories. There is a requirement for an efficient defense mechanism that can counter adversarial attacks from all categories. Therefore, to counter state-of-the-art adversarial attacks more systematically, the proposed research will focus on the development and evaluation of a defense mechanism that is more generic and is effective against state-of-the-art attack types. Such a versatile defense mechanism will be able to ensure the secure deployment of pneumonia detection deep learning models in practical scenarios.

## Materials and methods

This section contains dataset details, generated attack hierarchy, methodological steps and defense technique proposed in this study.

### Data set

A dataset [9] was compiled using publicly available pediatric chest X-rays (posteroanterior (PA) view) of children aged one to five years old, obtained from Guangzhou Women and Children’s Medical Center in Guangzhou, China, as part of their routine clinical care. This dataset, comprising 5,814 X-ray images, was utilized for training, and testing the model^1^.

**Fig 1:**
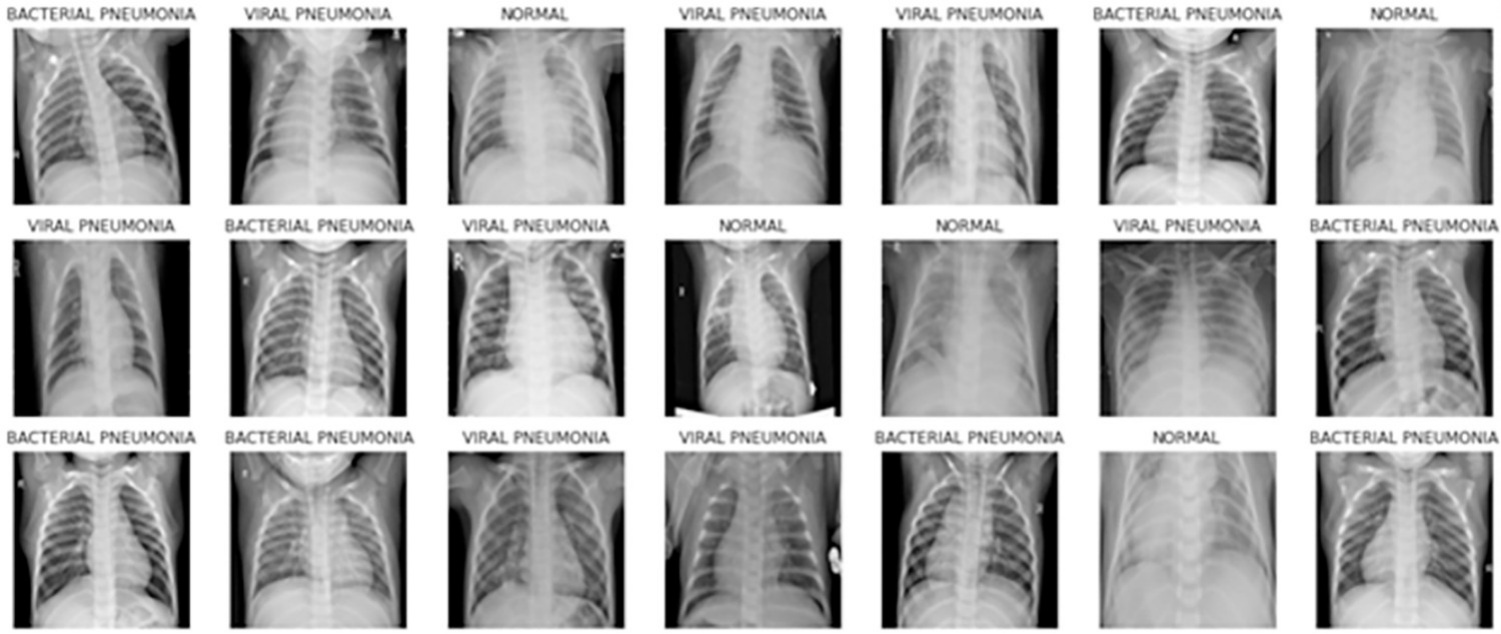
Sample images of dataset from bacterial, viral and normal classes.

The images fall into three categories: a) bacterial pneumonia, b) normal, and c) viral pneumonia (refer to Table 3). The dataset is organized into three folders: ’train’, ’test’, and ’validation’. It was split into an 80:20 ratio for training and validation purposes. All chest X-ray (CXR) images in the dataset are stored as JPEG files with dimensions of 224x224 pixels. Sample images are depicted in Fig 1.

**Table 3:** Total dataset for pretrained models.

Data	Normal	Bacterial Pneumonia	Viral Pneumonia	Total
Test	234	242	140	616
Train	1040	2024	1076	4140
Validation	260	506	268	1034
Total	1534	2772	1484	5790

### Generated attacks hierarchy

10 Attack folders were generated having (6750+6750 = 13500) total generated adversarial images, including 5 Attack folders for PGD and 5 Attack folders for FGSM (450 images x 5 epsilons x 3 classes = 6750 per Attack) respectively. Tables 4 and 5 represent PGD and FGSM Attack folders hierarchy.

**Table 4:** PGD attack generated images.

	Train	Test	Validation	Total
**No. of images**	250	100	100	450
**epsilons(0.001,0.006,0.05,0.1, 0.4)**	5	5	5	5
**No. of classes(Bacterial, viral, normal)**	3	3	3	3
**Total**	(250x5x3) = 3750	(100x5x3) = 1500	(100x5x3) = 1500	(450x5x3) = 6750

**Table 5:** FGSM attack generated images.

	Train	Test	Validation	Total
**No. of images**	250	100	100	450
**epsilons(0.001,0.006,0.05,0.1, 0.4)**	5	5	5	5
**No. of classes(Bacterial, viral, normal)**	3	3	3	3
**Total**	(250x5x3) = 3750	(100x5x3) = 1500	(100x5x3) = 1500	(450x5x3) = 6750

### Methodological steps

**Fig 2:**
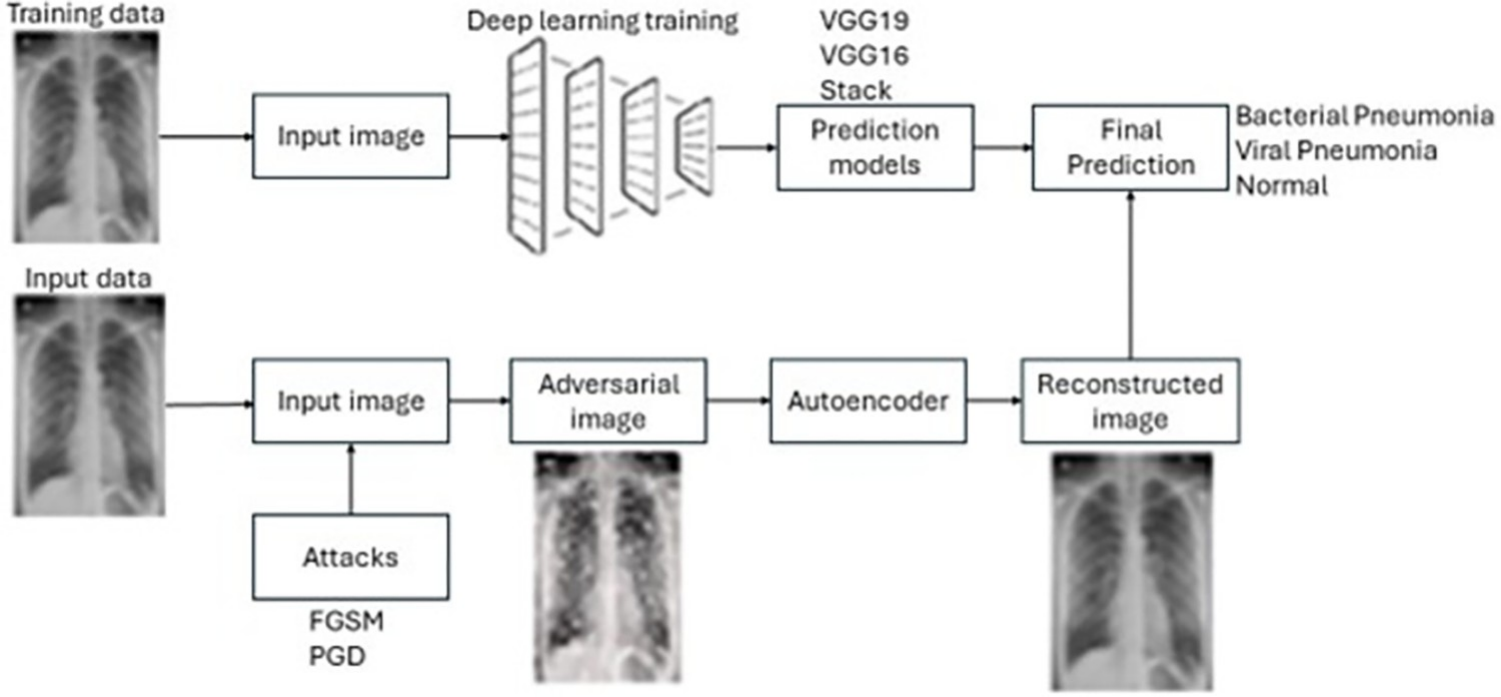
Methodology of proposed framework.

To enhance system robustness, we introduce a defense mechanism against adversarial attacks. In this approach, an auto-encoder is employed to preprocess images before their input into the classifier. The auto-encoder primarily denoises images by compressing them into a lower- dimensional space through its encoder component and then transfers the reconstructed image to the classifier. The Convolutional autoencoder takes FGSM and PGD perturbed images and converts them into reconstructed output. FGSM and PGD attacks are carried out at 5 magnitudes, i.e., ε (epsilon) values. For FGSM (0.001, 0.006, 0.05, 0.1, 0.4), for PGD (0.001, 0.006, 0.05, 0.1, 0.4). We have utilized two pre-trained models, VGG19 and VGG16, along with a hybrid model called Stack Model, which was created by merging two models, namely MobileNetV2 and DenseNet169. We then compared their results. The methodology of the proposed framework includes preprocessing of chest X-ray images, data augmentation (Rotation, Scaling, and Translation), feature extraction, auto-encoder as an adversarial detector, and classification as shown in Fig 2.

**Fig 3:**
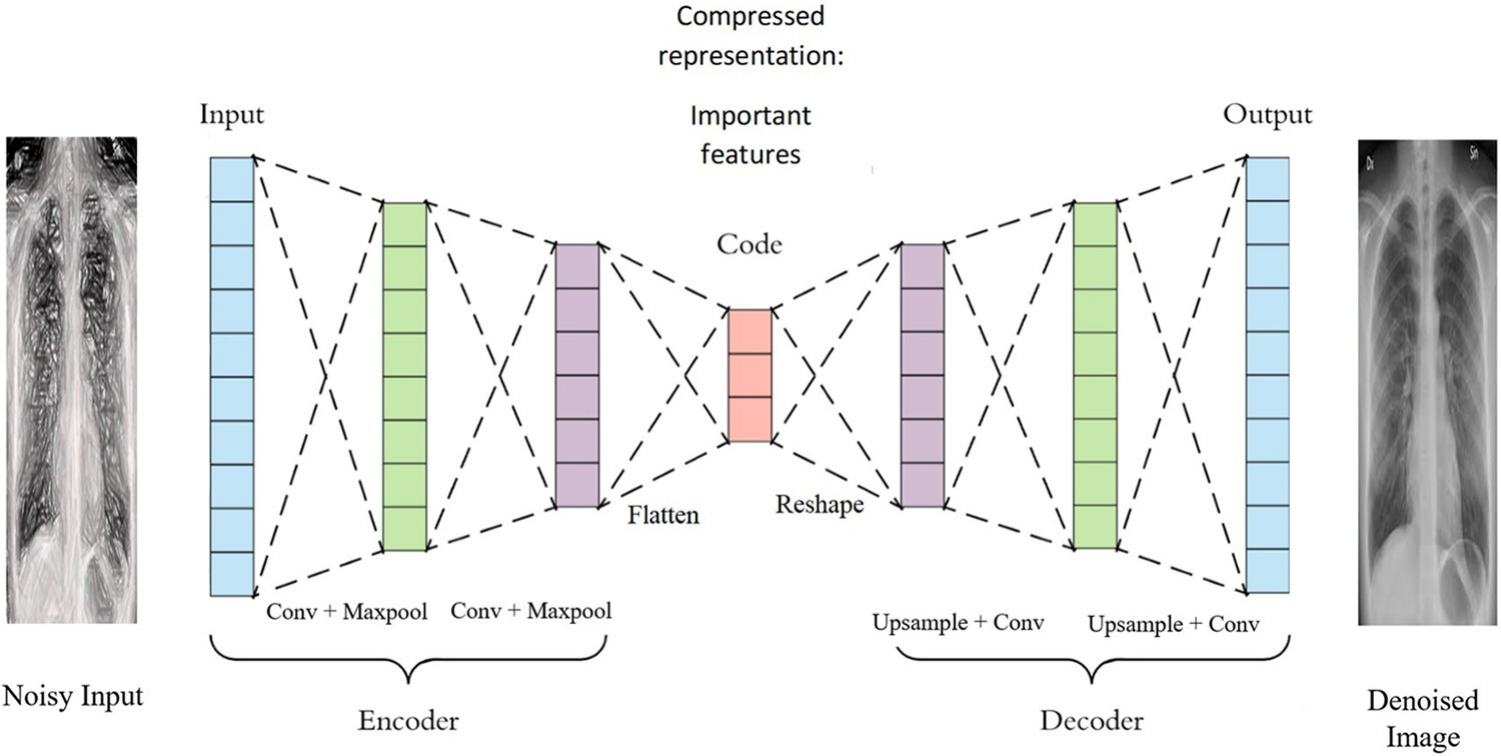
Convolutional Autoencoder [57].

A convolutional Autoencoder is like a standard auto-encoder, but the encoder and decoder use convolutional and pooling layers instead of dense layers. This is useful for processing data that has a grid-like structure, such as images. The encoder down samples the input image and the Decoder up samples the image. The structure of a convolutional auto-encoder [57] is shown in Fig 3.

Configuration of the proposed Auto-Encoder involves setting the architecture, defining hyperparameters, and specifying the training process. The architecture includes an Input Layer which match thedimensions of your input data. The encoder includes 2D convolutional layers with Maximum poolinglayers (subsampling) to reduce spatial dimensions. Decoder mirror the encoder architecture with upsampling instead of pooling using Convolution Transpose Layer and Convolutional Layers. (see Table 6). Filter Size which decides on the size of convolutional filters, i.e., 3x3. Set the striding parameters for down-sampling in the encoder. Use activation functions ReLU for hidden layers, and sigmoid for the output layer.

**Table 6:** Configuration of proposed Auto-encoder.

Type	Layer	Filter Size	Stride	Output Shape	# of parameters	Padding
Encoder	Input Layer	24x24	------	[(None, 224, 224, 1)]	0	------
Encoder	Convolution Layer	3x3	------	(None, 224, 224, 32)	320	Same
Encoder	ReLU	------	------	------	------	------
Encoder	Maximum Pooling Layer	2x2	------	(None, 112, 112, 32)	0	Same
Encoder	Convolution Layer	3x3	------	(None, 112, 112, 32)	9248	Same
Encoder	ReLU	------	------	------	------	------
Encoder	Maximum Pooling Layer	2x2		(None, 56, 56, 32)	0	Same
Decoder	Convolution Transpose Layer	3x3	2	(None, 112, 112, 32)	9248	Same
Decoder	ReLU	------	------	------	------	------
Decoder	Convolution Transpose Layer	3x3	2	(None, 224, 224, 32)	9248	Same
Decoder	ReLU	------	------	------	------	------
Decoder	Convolution Layer	3x3		(None, 224, 224, 1)	289	Same
Decoder	Sigmoid	------	------	------	------	Same

**Fig 4:**
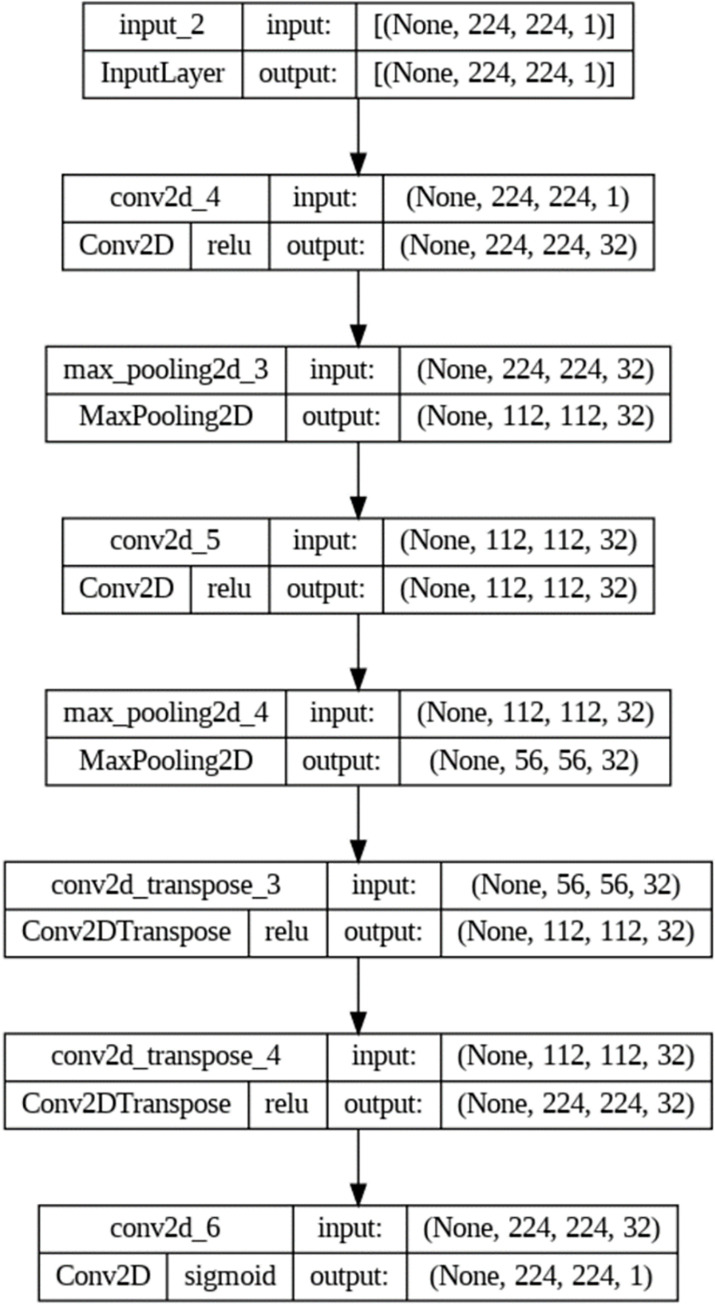
Layers representation of Auto encoder.

Fig 4 illustrates the layers of the Autoencoder, showcasing the Encoder which comprises Convolutional Layers responsible for applying convolutional operations to the input data. These convolutional filters learn hierarchical features within the input images. Additionally, the Encoder includes Pooling Layers, which downsample the spatial dimensions of the feature maps, reducing computation while preserving important features. Activation Functions are then applied to introduce non-linearity to the network, enabling it to capture complex relationships. The Latent Space is represented by a dense layer with a small number of neurons, serving as the compressed or encoded version of the input.

On the other hand, the decoder consists of Deconvolutional layers (Transpose Convolution) that upscale the feature maps back to the original input dimensions, facilitating the reconstruction of the input from the compact latent space. Upsampling layers, similar to pooling layers but in the opposite direction, increase the spatial dimensions of the feature maps. Lastly, the Output layer serves as the final layer, producing the reconstructed output.

Train the model using the input images as both input and target, aiming to minimize the reconstruction loss. Tables 7 and 8 shows training input of encoder taken from un-attacked (clean) dataset and decoder output taken from un-attacked (clean) datasets.

**Table 7:** Autoencoder input.

	Train images un- attacked(clean)	No. of Classes (Bacterial, viral, normal)	Total
**Input images**	1000	3	1000x3 = 3000

**Table 8:** Autoencoder output.

	Train images un- attacked(clean)	No. of Classes (Bacterial, viral, normal)	Total
**Output images**	1000	3	1000x3 = 3000

In the training process, choose an Optimizer for reducing the overall loss and improving accuracy. Batch Size that defines the number of samples in each batch. Epochs that decide on the number of training epochs. Loss Function is used for reconstruction loss i.e. measures the difference between the input and the reconstructed output.

As shown in Table 9, we selected Mean Square Error (MSE) as a Loss Function. MSE is straightforward to implement and compute. It measures the average squared difference between the reconstructed output and the original input. MSE loss is differentiable with respect to the model parameters, which is crucial for training using gradient-based optimization algorithms such as stochastic gradient descent (SGD) or its variants. This allows for efficient optimization through backpropagation. MSE loss can be interpreted as an estimate of the variance of the reconstruction error. Minimizing MSE effectively reduces the variance of the reconstruction error, which is often desirable in denoising tasks. In denoising tasks, preserving fine details and accurately reconstructing individual pixels is often important. MSE loss penalizes large errors heavily, which encourages the model to focus on minimizing the errors in individual pixels. Choose Adam (Adaptive Moment Estimation) as an Optimizer. Its adaptive learning rates, efficiency in training, low memory requirements, simple implementation, robustness to noisy gradients, and reduced sensitivity to hyper parameters make it a suitable choice for auto-encoder. We set the Learning Rate of 0.001 for Adam. It is a commonly chosen learning rate for Adam because of its empirical success, moderate Step Size, it is compatible with Adaptive Methods, its generalization performance, consistency across implementations. We chose Sigmoid as an Activation Function interpreted its output between 0 and 1, to ensure the output of the autoencoder is also within this range, facilitating easy comparison between input and output during training. Its non-linearity and smooth gradient feature make it the best choice. Select 30 epochs, because it balances training time and model performance. It is an Empirical observation in many cases that lead to satisfactory results in terms of reconstruction accuracy and feature learning. It is a part of a strategy that includes early stopping based on validation performance. By monitoring the validation accuracy on a validation set, training can be stopped if performance begins to deteriorate, indicating that the model is starting to overfit. A moderate number of epochs like 30 can help manage computational resources while still allowing for meaningful training. Consistency with prior work can facilitate comparisons with prior work and ensure reproducibility of results. A batch size of 128 allows for better utilization of GPU memory and processing power compared to smaller batch sizes. Larger batch size makes the gradient estimation tend to be more accurate since it’s based on more data points. This can lead to more stable training and faster convergence. Batch Size helps the model generalize better to unseen data by providing more diverse examples in each training iteration. With a larger batch size, the noise in the gradients caused by individual data points can be reduced, leading to smoother optimization trajectories. Larger batch sizes require more computation per iteration, they often result in fewer iterations needed for convergence, potentially speeding up the training process overall.

**Table 9:** The hyper parameters for the auto-encoder training.

Loss Function	Mean Square Error
**Optimizer**	Adam
**Learning Rate**	0.001
**Activation Function**	Sigmoid
**Number of epochs**	30
**Batch size**	128

**Fig 5:**
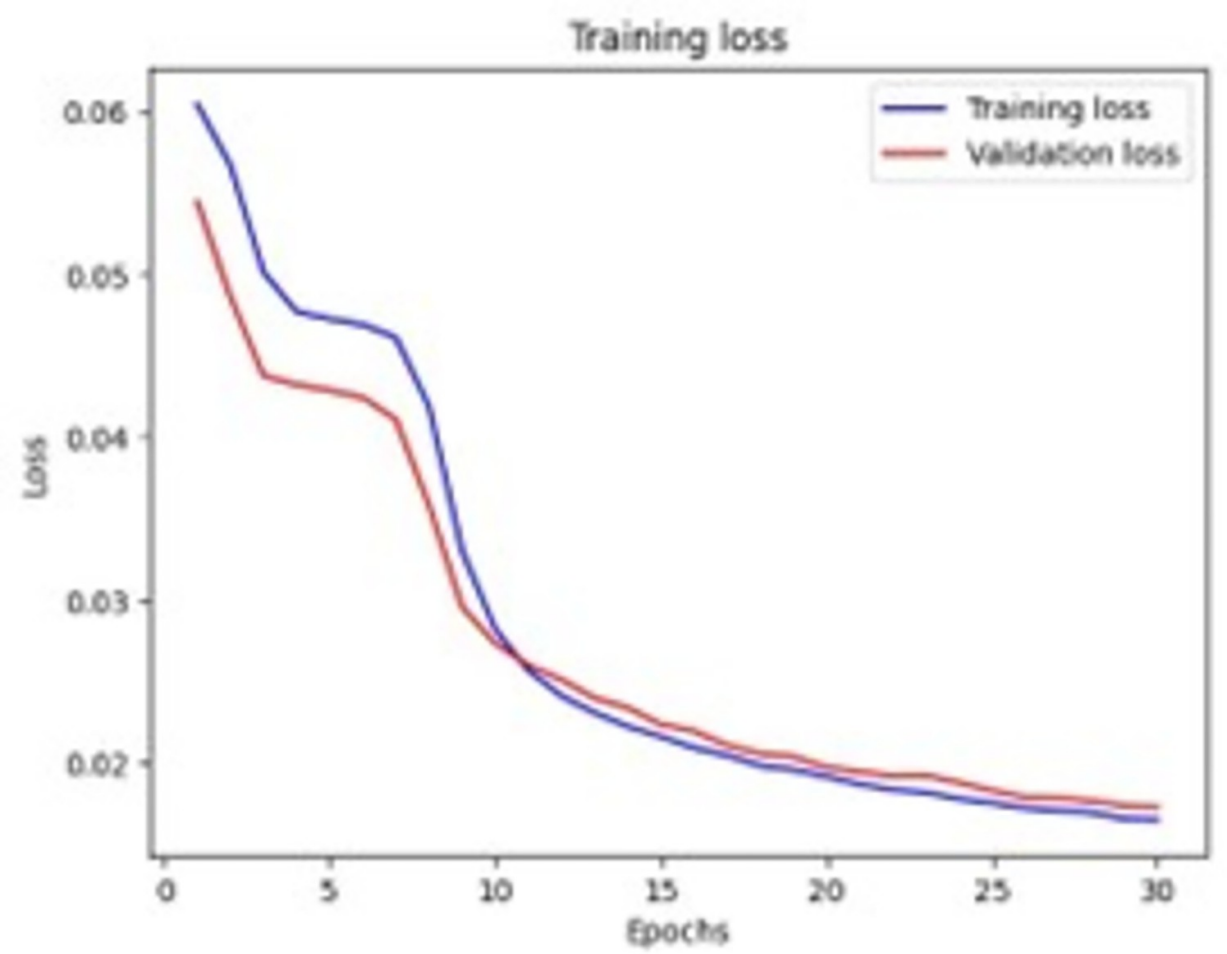
Convergence graph of autoencoder.

Fig 5 shows the performance of an autoencoder through convergence graph shows training loss 0.0165 and validation loss 0.0173.

We have utilized 2 pre-trained models VGG19 and VGG16 and merged two models i.e. MobileNetV2 and DenseNet169 to create a hybrid model called Stack Model.

**Fig 6:**
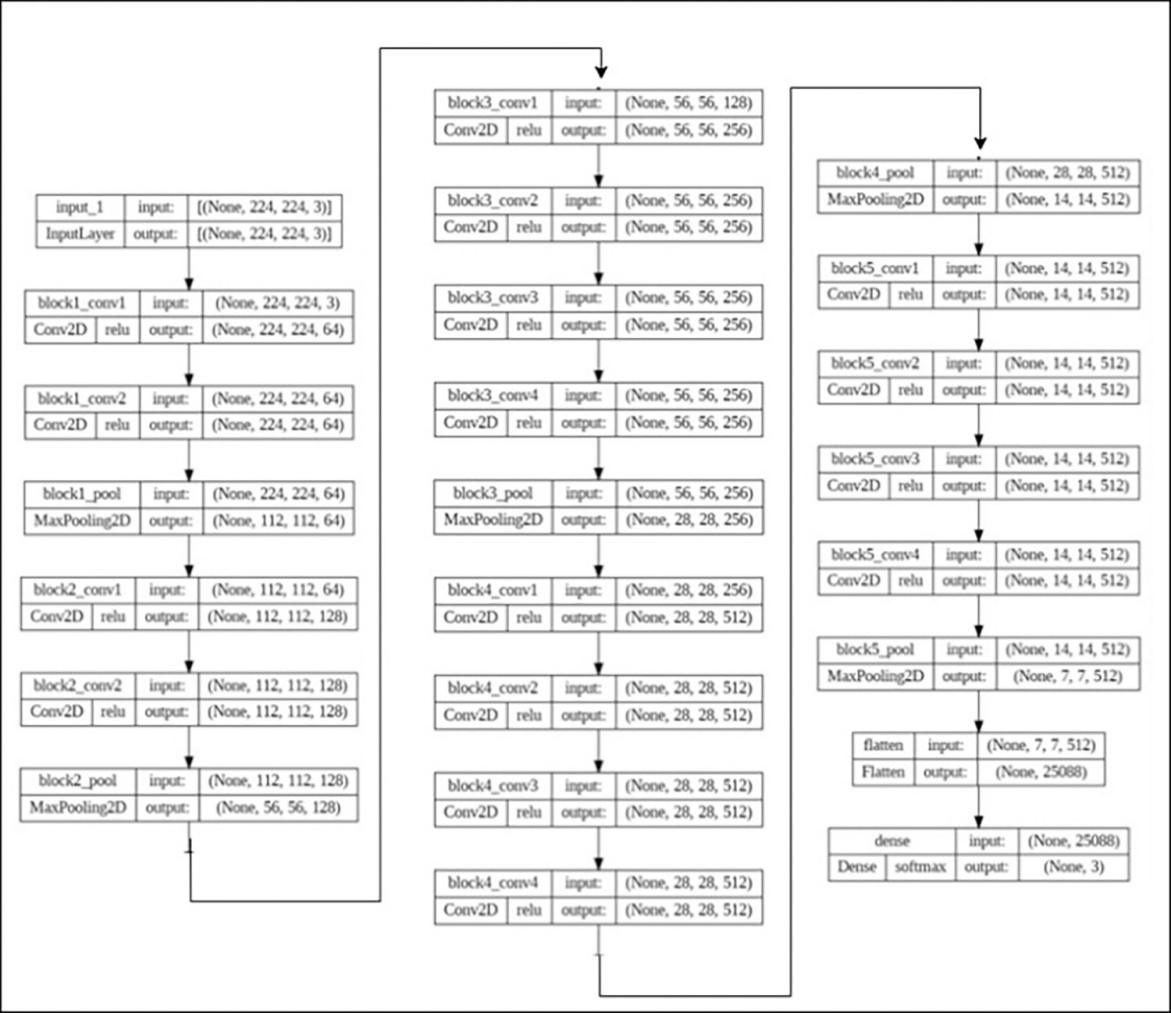
VGG19 layer representation.

**Fig 7:**
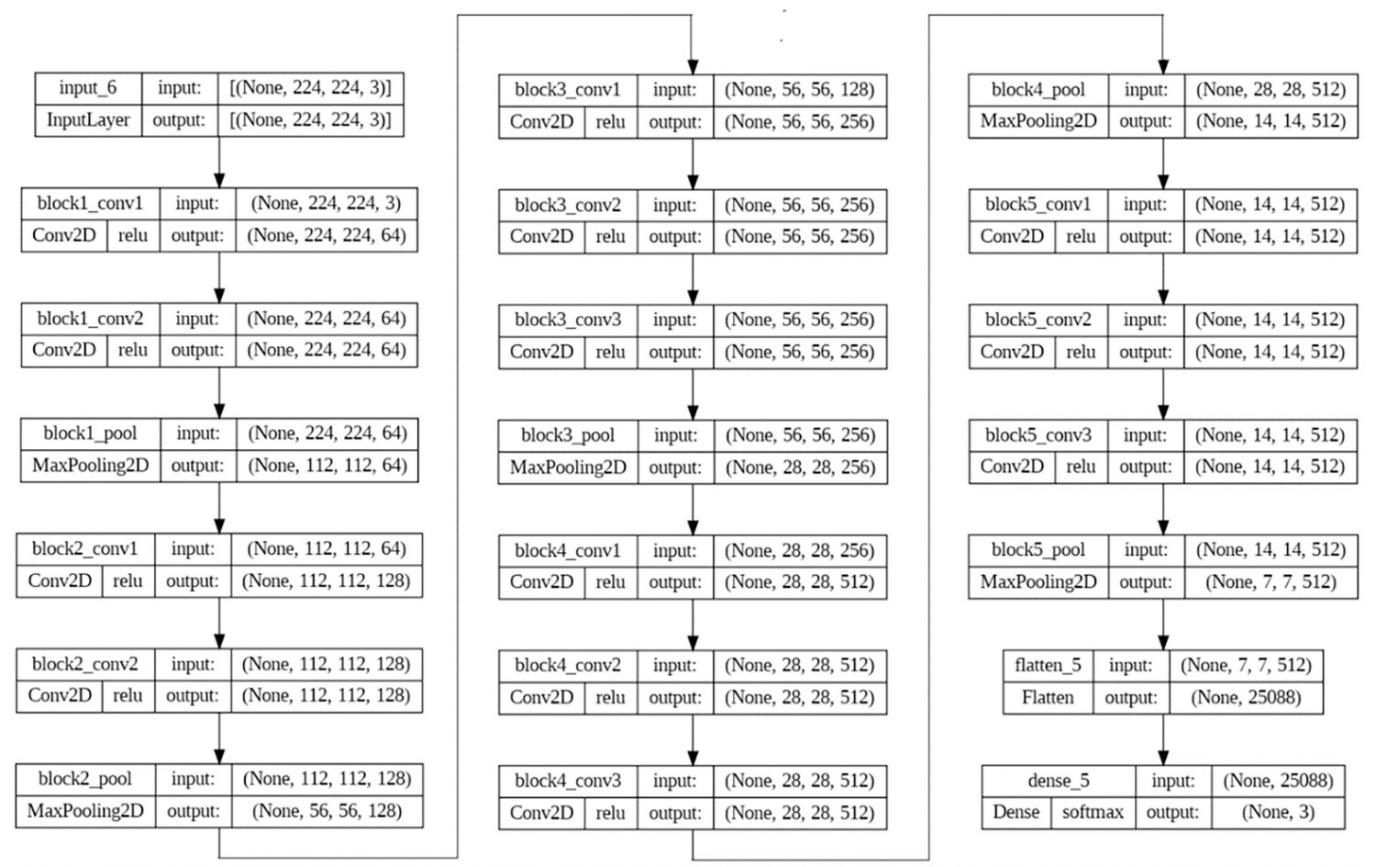
Layers of VGG16 model.

Figs 6 and 7 represents layers of VGG19 and VGG16 Model. Merging models in deep learning, often referred to as ensemble learning or model fusion, can offer several advantages: It allows combining their features, potentially leading to a more comprehensive representation of the data. It can also lead to better generalization performance on unseen data. Merging models in deep learning, commonly known as ensemble learning or model fusion, presents various benefits. It enables the combining of their features, potentially resulting in a more comprehensive representation of the data. Additionally, it can enhance generalization performance on unseen data. It can also exploit the strengths of each model to improve overall performance, such as accuracy, speed, or efficiency. Hybrid models trained on different domainsor datasets can help adapt a model to a new domain by transferring knowledge from both models. Merging them allows creating a specialized model tailored to the requirements of the task at hand. It can also lead to a more compact representation compared to using each model individually, which can be beneficial for deployment on resource-constrained devices or systems. Many deep learning chest X-ray image based detection researches have applied hybrid model approach [58–67]. We can explore novel architectures or combinations from that, aiming to push the boundaries of deep learning performance or capabilities.

**Fig 8:**
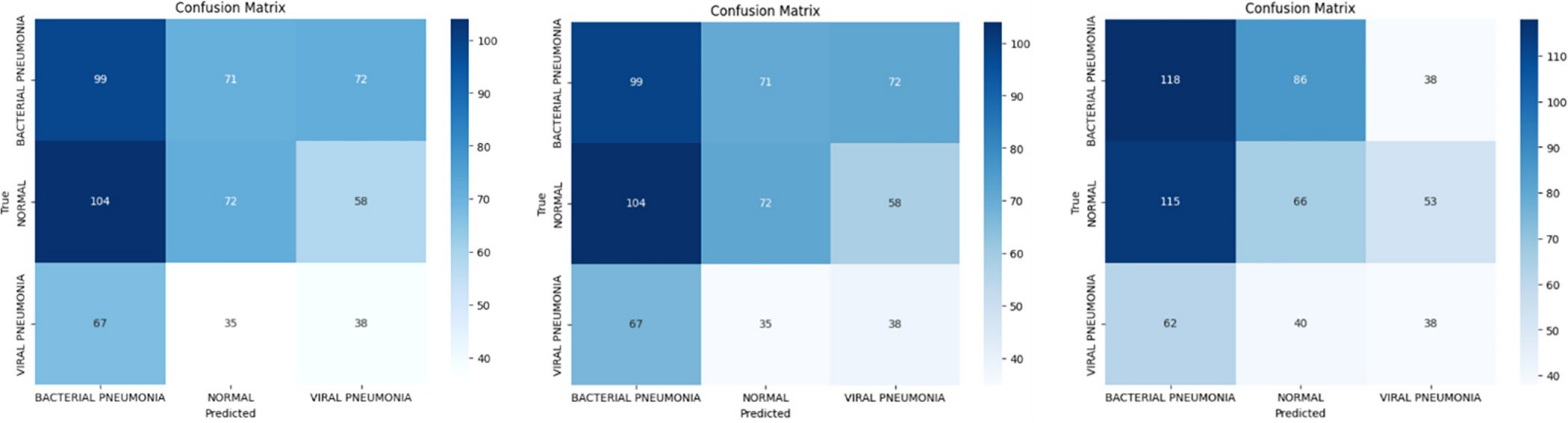
Confusion matrices where, (a) represent VGG 19, (b) represent VGG16 and (c) represent stack model.

Fig 8A–8C show the Confusion matrices of VGG19, VGG16 and Stack model performances respectively.

The hyper parameters during training and Layers configuration including output shape and number of parameters of Stack model are shown in Tables 10 and 11.

**Table 10:** The hyper parameters of the stack model.

**Loss Function**	Binary Cross Entropy
**Optimizer**	Adam
**Activation Function**	Softmax
**Learning Rate**	0.0001
**Number of epochs**	30
**Batch size**	21

**Table 11:** Stack model summary.

Layer	Output Shape	# of parameters
Input Layer	[(None, 224, 224, 3)]	0
mobilenetv2_1.00_224 (Functional)	(None, 7, 7, 1280)	2257984
densenet169 (Functional)	(None, 7, 7, 1664)	1264288
global_average_pooling2d(GlobalAveragePooling2D)	(None, 1280)	0
global_average_pooling2d_1(GlobalAveragePooling2D)	(None, 1664)	0
flatten (Flatten)	(None, 1280)	0
flatten_1 (Flatten)	(None, 1664)	0
concatenate (Concatenate)	(None, 2944)	0
batch_normalization (Batch Normalization)	(None, 2944)	11776
dense (Dense)	(None, 256)	753920
ReLU	------	------
dropout (Dropout)	(None, 256)	0
batch_normalization_1 (BatchNormalization)	(None, 256)	1024
dense_1 (Dense)	(None, 128)	32896
ReLU	------	------
dropout_1 (Dropout)	(None, 128)	0
dense_2 (Dense)	(None, 3)	387
Softmax	------	------

This study suggested a defense strategy to make the model’s diagnosis more robust, through the addition of an auto-encoder on the CNN structure. The proposed technique can be applied on chest X-ray images, including FGSM, PGD adversarial examples.

## Results and discussions

### Methods of evaluation

To evaluate the results, we employed Accuracy, which measures the overall performance of a model. Additionally, we utilized the confusion matrix to visualize the performance of the models. Recall (Sensitivity), or True Positive Rate (TPR), quantifies the number of positive cases correctly predicted by the model. Precision is a metric that assesses the model’s accuracy in predicting positive samples. F1-Score represents the harmonic mean of precision and recall. Finally, the results will be compared to the seven baseline studies conducted in the same modality, task, and method of attack.

### Experimental results and discussion

This section describes the performance of the 3 target models VGG16, VGG19, and Stack model subjected to adversarial samples FGSM and PGD attacks using 10 Attack intensities, in the absence and presence of an Auto-encoder.

### FGSM data

**Fig 9:**
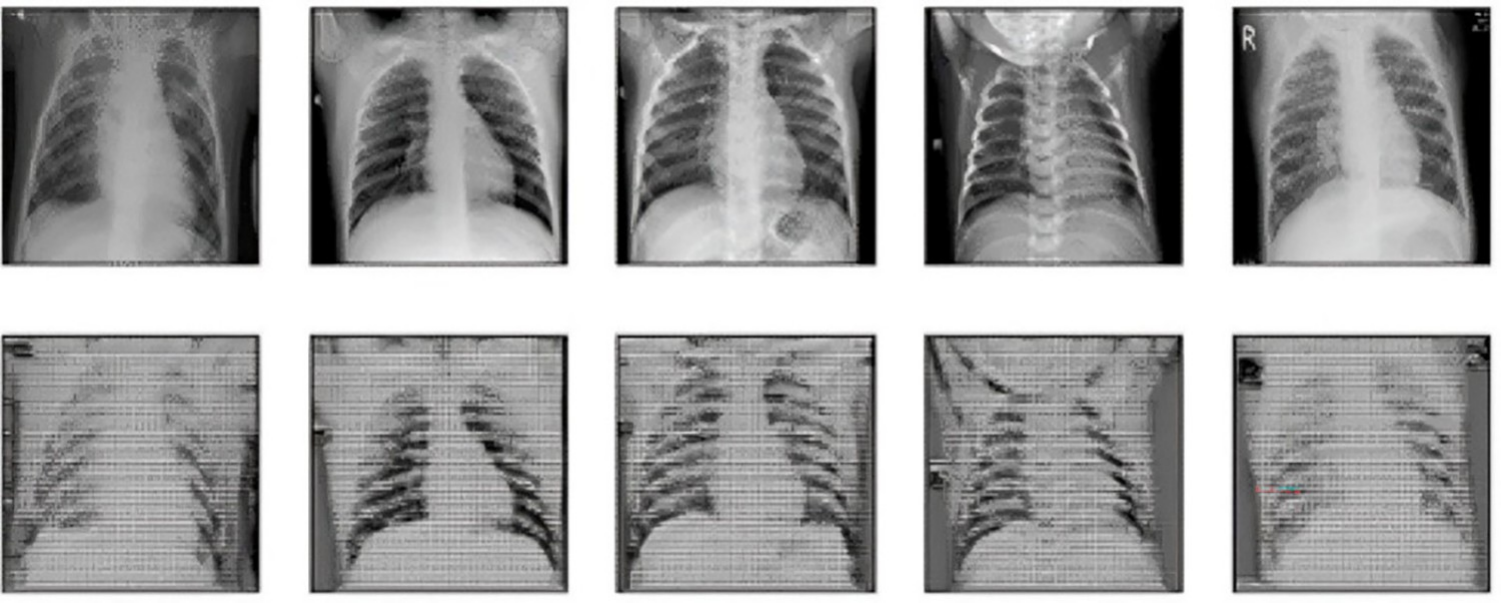
FGSM perturbed images at epsilon 0.1. The First row represents the original and the second row represents reconstructed images at the Combine Auto-encoder.

Fig 9 shows FGSM perturbed images at epsilon *∊* 0.1. Represents original and reconstructed images at an Auto Encoder.

**Fig 10:**
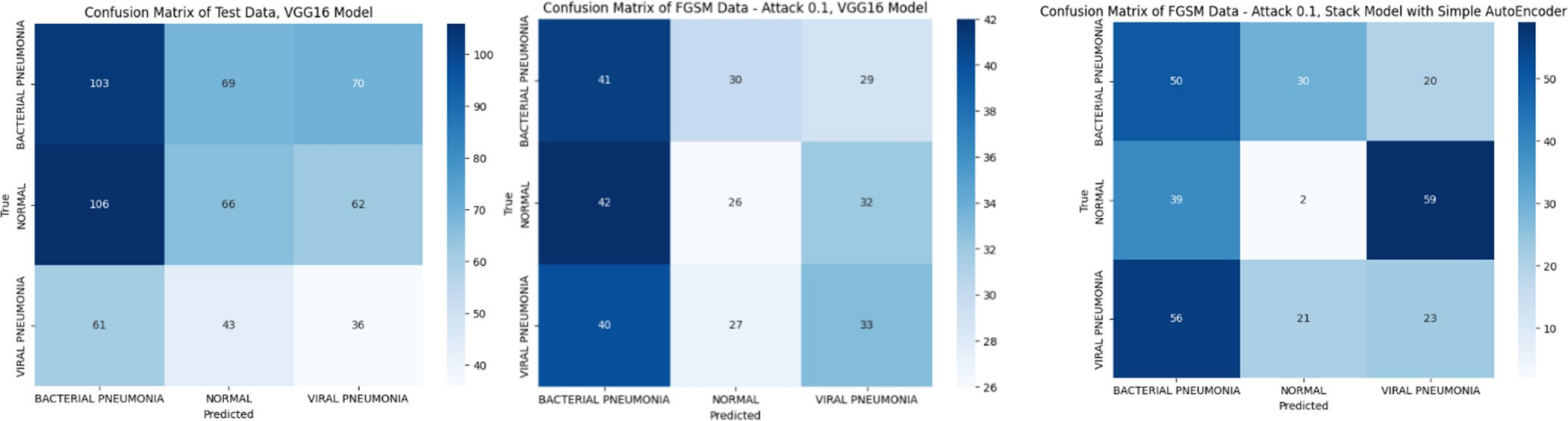
Shows Confusion Matrix of FGSM data at epsilon 0.1, where (a) represents, Test Data before attack, (b)represents, FGSM Data—Attack, VGG16 Model, (c) represents FGSM Data—Attack, Stack Model with AutoEncoder.

Results in Fig 10 show how fairly models (VGG19, VGG16, and Stack models) can distinguish between the 3 classes visualizing Confusion Matrices with respected AUC ROC curves.

### PGD data

**Fig 11:**
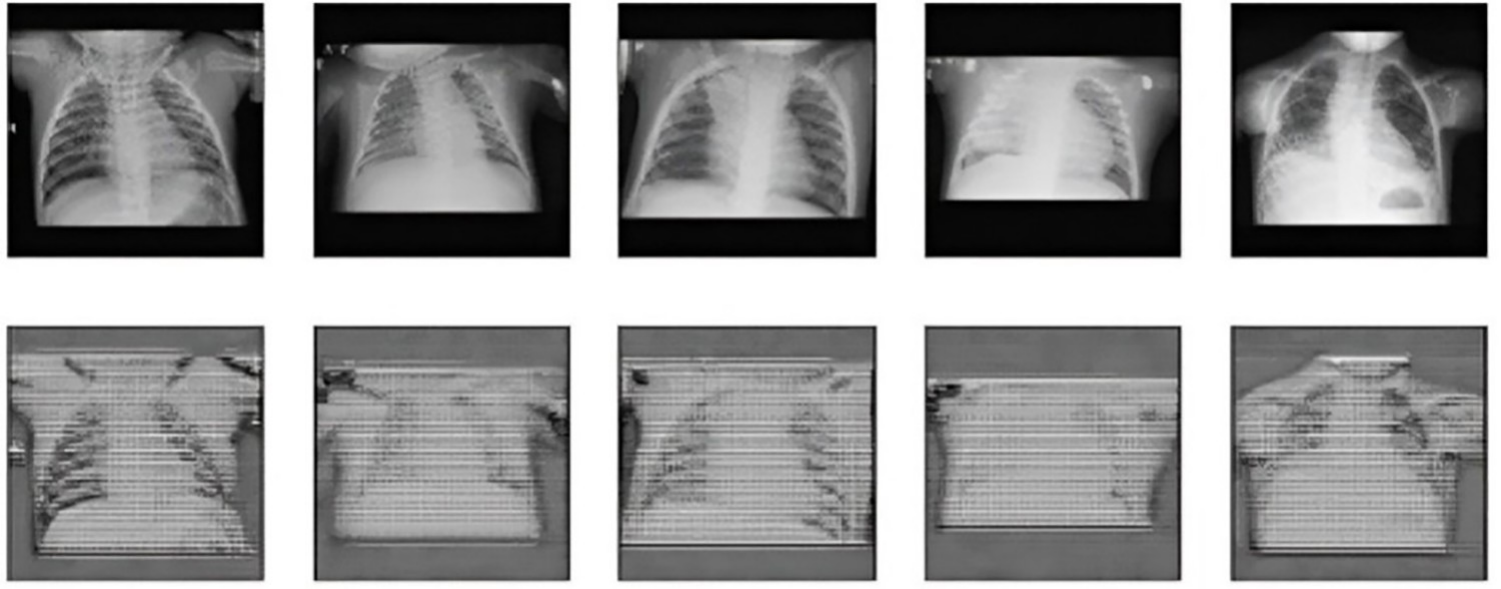
PGD perturbed images at epsilon 0.05. First row represents original, and second row represents reconstructed images at an Auto-encoder.

Fig 11 shows PGD perturbed images at epsilon *∊* 0.05. Represents original and reconstructed images at an Auto Encoder.

**Fig 12:**
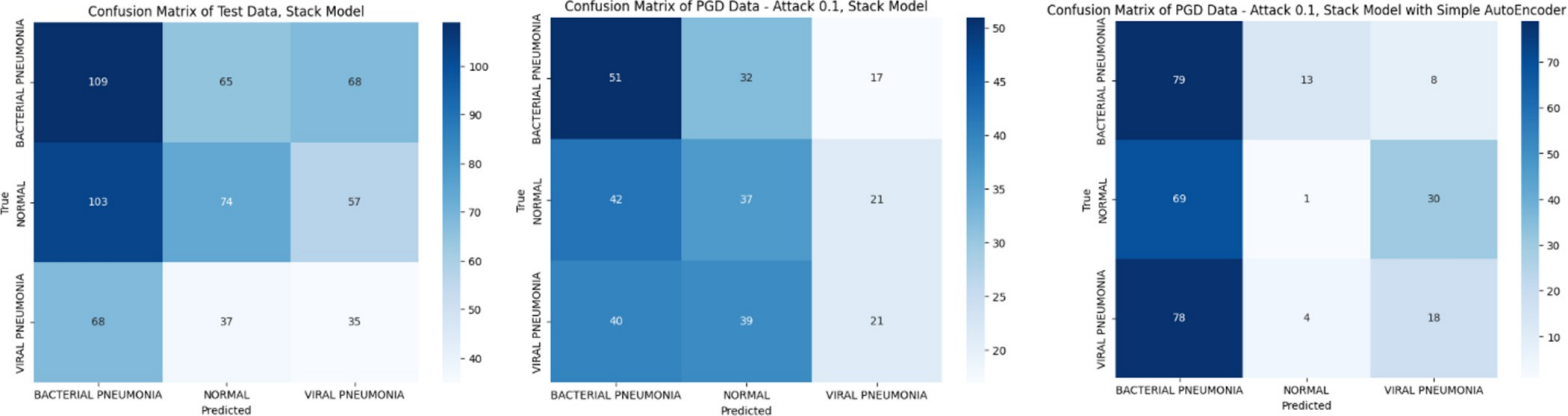
Shows Confusion Matrix of PGD data at epsilon 0.1, where (a) represents, Test Data before attack, represents, PGD Data—Attack, Stack Model, (c) represents PGD Data—Attack, Stack Model with AutoEncoder.

Results in Fig 12 show how fairly models (VGG19, VGG16 and Stack models) can distinguish between the 3 classes visualizing Confusion Matrices with respected AUC ROC curves.

### FGSM and PGD attack performance

Table 12 presents Accuracy before and after FGSM and PGD Attacks. VGG19, VGG16 and Stack Model are validated on the test set (clean samples) included in the Chest X-ray dataset and it achieves an accuracy of 84%, 86% and 82.6%. Maximum Accuracy drop for FGSM Attack is up to 50%, 55% and 54% for VGG19, VGG16 and Stack model respectively. Thus, VGG19 is more resilient than Stack and VGG16 and shows a low accuracy drop. However, Maximum Accuracy drop for PGD Attack is up to 53%, 67% and 52.6% for VGG19, VGG16 and Stack model respectively. Therefore, the Stack Model is more resilient than VGG19 and VGG16.

**Table 12:** Accuracy before and after FGSM and PGD attacks.

		VGG19			VGG16			Stack Model		
Samples		Accura cy	Accurac y Drop	Attack Succes s Rate	Accurac y	Accurac y Drop	Attack Succes s Rate	Accurac y	Accurac y Drop	Attack Succes s Rate	
**Clean**		84%	------		86%	------		82.6%	------		
	ε **= 0.00**	38%	46%	62%	31%	**55%**	69%	28%	**54.6%**	72%	
	**1**										
	ε **= 0.00**	35%	49%	65%	35%	51%	65%	34%	48.6%	66%	
**FGSM**	**6**										
	ε **= 0.05**	36%	48%	64%	35%	51%	65%	33%	49.6%	67%	
	ε **= 0.1**	34%	**50%**	66%	33%	53%	67%	37%	45.6%	63%	
	ε **= 0.4**	37%	47%	63%	31%	**55%**	69%	34%	48.6%	48.6%	
**PGD**	ε **= 0.0 01**	36%	48%	64%	31%	**55%**	69%	31%	**51.6%**	69%	
	ε **= 0.0 06**	38%	46%	62%	35%	51%	65%	30%	**52.6%**	70%	
	ε **= 0.05**	34%	50%	66%	33%	**67%**	67%	30%	**52.6%**	70%	
	ε **= 0.1**	31%	**53%**	69%	37%	49%	63%	36%	46.6%	64%	
	ε **= 0.4**	31%	**53%**	69%	30%	**56%**	70%	32%	**50.6%**	68%	

The results in Table 12 show a drastic decrease in the overall accuracy values when performing attacks in PGD using VGG16 model. The Precision, recall (sensitivity) and F1 score in Table 14 also confirm this observation.

**Fig 13:**
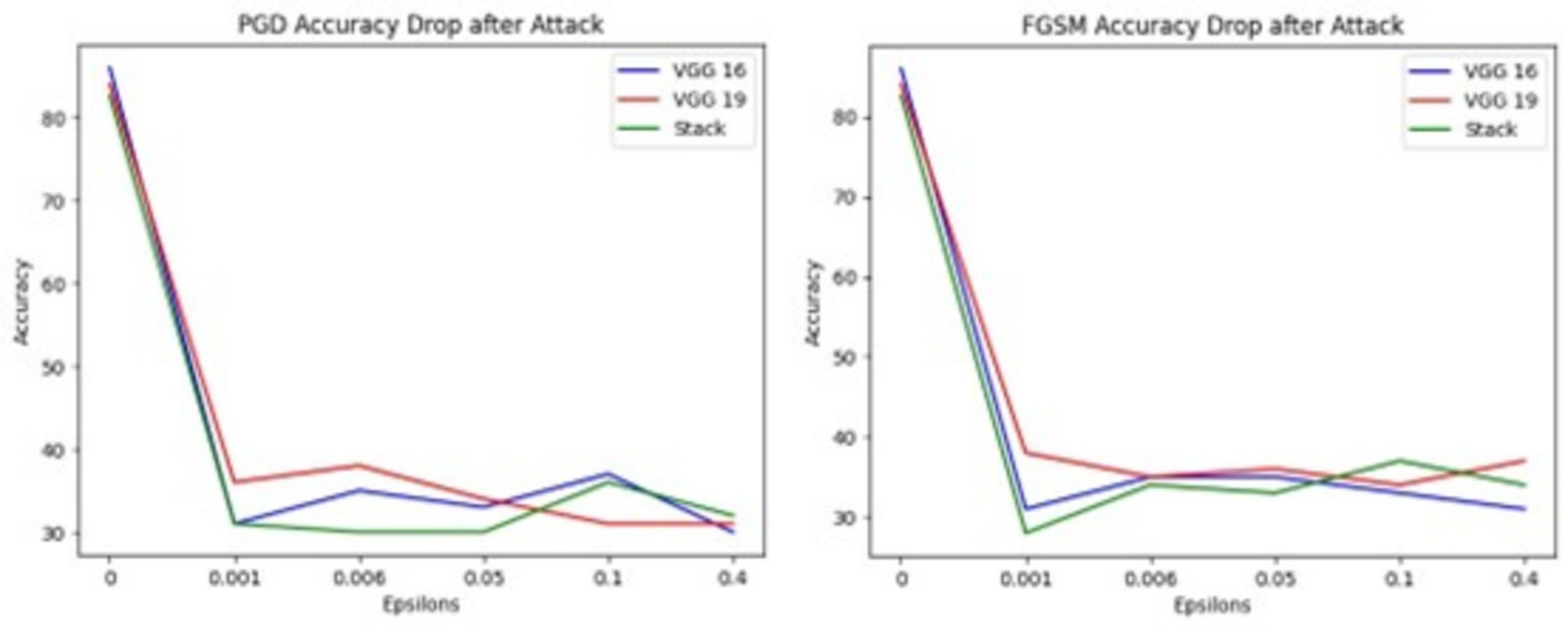
Accuracy before and after Attacks, where (a) represents accuracy drop after PGD Attack (b) represents accuracy drop after PGD attack.

Fig 13 shows graphical representations of accuracy before and after attacks maximum accuracy drop ranges from 84% to 33% in VGG16, VGG19 and Stack model.

Table 13 represents Precision, recall (sensitivity) and F1 score values before and at the time of Attacks using Autoencoder with 10 Attack epsilon values on 3 target models VGG19, VGG16 and Stack models. As we have class imbalance, we selected weighted average of 3 classes, Weighted average considers the class distribution in the dataset.

**Table 13:** Precision, recall (sensitivity) and F1 score before and after FGSM and PGD attacks.

Samples		VGG19			VGG16			Stack Model		
		Precisi on	Recall	F1 Score	Precisio n	Recall	F1 Score	Precisio n	Recall	F1 Score
**Clean**		0.37	**0.38**	0.37	0.34	0.33	0.33	**0.34**	0.35	0.34
**FGSM**	ε **= 0.00** **1**	0.39	0.38	0.38	0.31	0.31	0.31	0.28	0.28	0.27
	ε **= 0.00** **6**	0.35	0.35	0.35	0.34	0.35	0.34	0.33	0.34	0.33
	ε **= 0.05**	0.36	0.36	0.35	0.36	0.35	0.35	0.33	0.33	0.32
	ε **= 0.1**	0.34	0.34	0.33	0.33	0.33	0.33	0.37	0.37	0.36
	ε **= 0.4**	0.37	0.37	0.37	0.31	0.31	0.31	0.34	0.34	0.33
**PGD**	ε **= 0.0 01**	0.38	0.36	0.31	0.33	0.31	0.30	0.30	0.31	0.30
	ε **= 0.0 06**	0.37	0.38	0.33	0.39	0.35	0.34	0.30	0.30	0.29
	ε **= 0.05**	0.29	0.34	0.28	0.35	0.33	0.32	0.30	0.30	0.29
	ε **= 0.1**	0.27	0.31	0.26	0.38	0.37	0.35	0.36	0.36	0.35
	ε **= 0.4**	0.29	0.31	0.26	0.30	0.30	0.28	0.32	0.32	0.31

### Autoencoder

Table 14 presents Accuracy with Defense using Auto-encoder. Maximum Accuracy improvement for FGSM Attack is up to 11%, 13% and 6% for VGG19, VGG16 and Stack model respectively. It has also been observed that Stack model fails to improve accuracy at epsilon 0.1 and shows -4 score (accuracy drop) at FGSM Attack. However, Maximum Accuracy improve for PGD Attack is up to 16%, 16% and 15% for VGG19, VGG16 and Stack model respectively.

**Table 14:** Accuracy with defense using autoencoder.

Defense Architecture		VGG19		VGG16		Stack Model	
		Accuracy	Accuracy Improve	Accuracy	Accuracy Improve	Accuracy	Accuracy Improve
Auto encoder							
**FGSM**	ε **= 0.001**	42%	4%	41%	10%	31%	3%
	ε **= 0.006**	42%	7%	41%	6%	37%	3%
	ε **= 0.05**	43%	7%	43%	8%	39%	**6%**
	ε **= 0.1**	45%	**11%**	46%	**13%**	33%	**-4%**
	ε **= 0.4**	45%	8%	44%	**13%**	40%	6%
**PGD**	ε **= 0.001**	45%	9%	46%	5%	46%	**15%**
	ε **= 0.006**	43%	5%	46%	11%	44%	14%
	ε **= 0.05**	49%	15%	45%	12%	43%	13%
	ε **= 0.1**	46%	15%	44%	7%	44%	8%
	ε **= 0.4**	47%	**16%**	46%	**16%**	38%	6%

**Fig 14:**
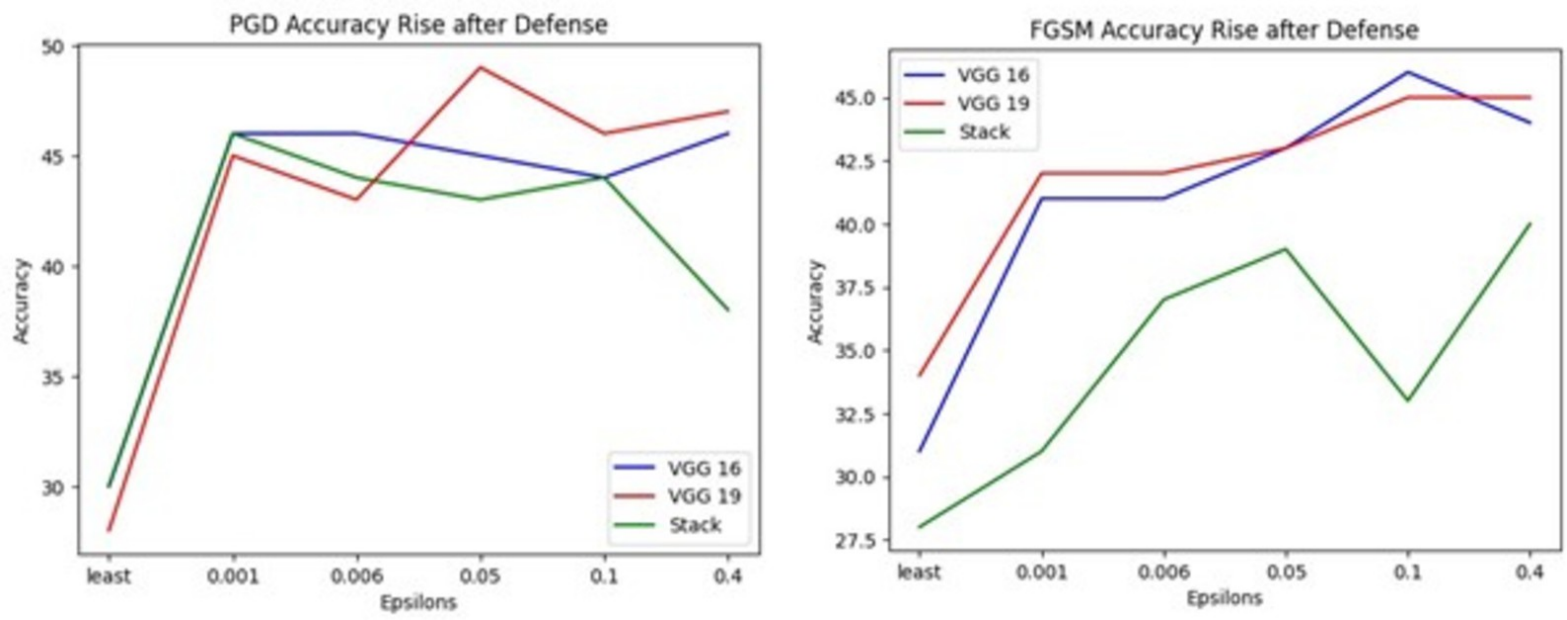
Accuracy after autoencoder defense, where (a) represents accuracy rise in PGD attack (b) represents accuracy rise in FGSM attack.

Fig 14 shows graphical representations of accuracy after autoencoder defense in which maximum accuracy rise ranges from 28% to 47% in VGG16, VGG19 and Stack model. Observe Fig 14A Green curve represents stack model failure at epsilon 0.1 shows accuracy drop (-4%).

### Comparative analysis

Table 15 discusses seven baseline studies in which model performance is examined after FGSM and PGD attacks in black-box settings at the modality of chest-x-ray images performed on the classification task. Results of our study have been compared with the performance of attacks and defenses through overall Accuracy drop at the time of Attack and Accuracy improved after Defense.

**Table 15:** Comparison with state-of-the-art studies.

Study	Attack performance		Defense performance	
	Attacks	Accuracy Drop	Accuracy Improve	Defense
[33], 2021	FGSM	Up to 7.41%	—	—
[29], 2022	FGSM	upto 23%	0% (black-box)	(HGD): Preprocessing PGL
	PGD	upto 20%	-31% -26%	LGL PGL+LGL
[53], 2022	FGSM	upto 52.5%	Absolute accuracy improves 3.7%	Adversarial Training
	PGD	upto 52.86%		
[40], 2021	FGSM	upto 41.1% avg	—	—
	PGD	upto 25.5% avg	—	—
[46], 2020	PGD	CXR: upto 41.5% avg;	upto 45%	FIUT images
			upto 44.5%	discretization-Based Transformed
	FGSM	CXR: upto 49.32% avg	upto 37%	FIUT images
			upto 36.5%	discretization-Based Transformed
[37], 2018	FGSM	from 20 to 24% (MP) from 13% to 20% (AP)	upto 9%	Feature Enhancement
	PGD	from 22 to 27% (MP) from 14% to19% (AP)		
[35], 2023	PGD	upto 62.82%	—	—
Our Study	FGSM	Up to **55%**	Up to **13%**	Auto Encoder
	PGD	Up to **67%**	Up to **16%**	Auto Encoder

Pal B et al. [33] investigated the classification accuracy of COVID-19 using X-rays and CT scans, applying the FGSM attack to generate adversarial samples. We select the diagnostic performance drop score resulting from the FGSM attack on chest X-ray images. These samples were then tested on both the VGG- 16 and InceptionV3 models. The VGG-16 model revealed the lowest reduction in accuracy of up to 7.41%, highlighting the vulnerability of this model.

Kansal K et al. [29] evaluated the adversarial robustness of COVID-19 classifiers by performing adversarial attacks: the Fast Gradient Sign Method (FGSM) and Projected Gradient Descent (PGD). The High-Level Representation Guided Denoiser (HGD) architecture has been assessed as a potential defensive technique for medical image analysis. Experiments were conducted in both white-box and black-box settings. We selected scores from the black-box setting, considering accuracy before and after respective attacks, which showed an accuracy drop of up to 23% in FGSM and 20% in PGD attacks. Accuracy scores with defenses were as follows: 0% increase in accuracy with the Pixel Guided Loss (PGL) architecture, - 31% increase in accuracy with Logit Guided Loss (LGL), and -26% increase in accuracy with a combination of PGL and LGL architectures. Therefore, in the black-box setting, the defense completely fails to protect against adversarial samples.

Joel MZ et al. [53] investigated the resilience of DL models trained on diagnostic images, including CT, mammogram, MRI, MNIST, and CIFAR-10, against adversarial attacks FGSM, PGD, and BIM. We selected scores showing the effects of adversarial attacks on model classification accuracy in mammogram images. The maximum accuracy dropped by up to 52.5% when applying the FGSM attack, and there was a 52.86% drop in accuracy in the case of PGD attack. After performing adversarial training, the DL model’s absolute accuracy increased by 3.7% for mammogram images.

Gougeh RA [40] examined the susceptibility of five neural networks—specifically ResNet-18, ResNet-50, Wide ResNet-16-8 (WRN-16-8), VGG-19, and Inception v3—to adversarial attacks. For this assessment, four distinct adversarial attack techniques, including the Fast Gradient Sign Method (FGSM), Projected Gradient Descent (PGD), Carlini and Wagner (C&W), and Spatial

Transformations Attack (ST) were utilized. Average accuracy drops up to 41.1% in the case of

an FGSM attack and 25.5% in the case of a PGD attack.

Tripathi AM et al. [46] presented a novel Fuzzy Unique Image Transformation (FUIT) technique that defends COVID-19 deep models trained on chest X-ray and CT image datasets against six adversarial attacks (FGSM, BIM, PGD without random start, PGD-r with random start, Deep Fool, C&W) by downsampling image pixels to an interval before training. Considering COVID-19 chest X-ray dataset scores, the average accuracy drop is 49.32% in the case of the FGSM attack and 41.5% in the PGD attack. For defense, using the FUIT-transformed technique in the case of the FGSM attack, accuracy increases by up to 37%, and using the discretization-based transformed technique, accuracy increases by up to 36.5%. In the case of the PGD attack, using the FUIT- transformed technique, accuracy increases by up to 45%, and using the discretization-based transformed technique, accuracy increases by up to 44.5%.

Asgari Taghanaki S et al. [37] attempted to replace max pooling with average pooling. They crafted adversarial examples using the InceptionResNetV2 and Nasnet Large models on a chest X-ray dataset, employing ten different attacks categorized into gradient-based, score-based, and decision- based attacks. In the case of an FGSM attack, accuracy drops from 20% to 24% with max pooling and from 13% to 20% with average pooling. In the case of a PGD attack, accuracy drops from 22% to 27% with max pooling and from 14% to 19% with average pooling. After performing feature enhancement, accuracy increases by up to 9%.

Siddiqi R et al. [35] examined the susceptibility of a pediatric pneumonia detection model to PGD attacks and observed a maximum accuracy drop of up to 62.82%.

By comparing the above seven studies, our study outperforms the research [29,33,35,37,40,46, 53] in terms of attack performance but shows vulnerability to FGSM and PGD attacks with a high accuracy drop. Similarly, our study outclasses the research [29,37,53] in terms of defense performance and shows robustness with improved accuracy.

## Conclusion

Deep learning has enhanced medical image analysis and has become an essential tool for medical experts. However, adversarial attacks obstruct the accurate operation of DL models and pose severe threats to patients. The proposed model will detect pediatric pneumonia in chest X-ray images using a robust classifier that is not vulnerable to state-of-the-art adversarial attacks, which are serious threats to the accurate prediction of medical diagnoses. To assess the model’s robustness, popular adversarial attacks FGSM and PGD for image classification will be generated. To counter adversarial attacks, we propose a convolutional autoencoder defense. The VGG16 model outperforms the other two models, demonstrating a better attack success rate with up to a 67% accuracy drop. The VGG19 and VGG16 models show defense success by improving accuracy by up to 16% against PGD attacks using the autoencoder. Our study shows satisfactory performance and outperforms state-of-the-art studies.

Despite many studies conducted on crafting adversarial attacks and their defense techniques, there is a need for more practical and effective defense methods to enhance the adversarial robustness of medical systems. While convolutional autoencoders can provide a level of defense against adversarial attacks, they are not a silver bullet. Adversarial attacks are an ongoing research area, and attackers continually develop new techniques. Therefore, a combination of approaches, including convolutional autoencoders, is often recommended for robust adversarial defense. The effectiveness of a defense strategy can vary depending on the specific use case and the nature of the adversarial attacks encountered. Regular updates and improvements to defense mechanisms are necessary to stay ahead of evolving threats.

In the future, these proposed models and their resilience can be tested on other medical datasets and against other state-of-the-art adversarial attack types. More chest diseases, such as lung mass (lung nodule) and tuberculosis, can be diagnosed. This research can be expanded to other modalities like CT scans, MRI, ultrasound, microscopy, and PET, as well as different target organs or tissues like the brain, liver, skin, and heart.
